# Investigation of Spexin Effects on Insulin‐Dependent Pathway Genes in High‐Glucose‐Induced Insulin‐Resistant C2C12 Myotubes

**DOI:** 10.1096/fba.2026-00003

**Published:** 2026-06-23

**Authors:** Ann Sze Cheah, Ji Wei Tan, Faizul Jaafar, Wendy Wai Yeng Yeo

**Affiliations:** ^1^ School of Science Monash University Malaysia Bandar Sunway Malaysia; ^2^ Department of Biochemistry, Faculty of Medicine Universiti Kebangsaan Malaysia Kuala Lumpur Malaysia; ^3^ School of Pharmacy Monash University Malaysia Bandar Sunway Malaysia

**Keywords:** C2C12 cells, diabetes, *GLUT4*, insulin resistance, skeletal muscle, spexin

## Abstract

Diabetes is a global health concern, with type 2 diabetes mellitus (T2DM) being the most common form. T2DM is characterized by insulin resistance and impaired glucose uptake in skeletal muscle. Spexin shows hypoglycaemic effects, but its mechanism of action, especially in skeletal muscles, remains elusive. This study aims to investigate the effects of spexin on key genes associated with the insulin‐dependent pathway in C2C12 cells. The differentiation of C2C12 myoblasts to C2C12 myotubes was confirmed using the fusion index and gene expression of myogenic factor 5 (*Myf5*), myosin heavy chain (*MyHC*), and myogenic regulatory factor 4 (*MRF4*) analysis. Cytotoxicity of spexin at varying concentrations (100 nM, 200 nM, 400 nM, 800 nM, and 1000 nM) was assessed via MTS assay. Insulin resistance–like conditions were induced in C2C12 myotubes using a high‐glucose medium and characterized at the mRNA level by quantitative reverse transcription PCR (RT‐qPCR) of peroxisome proliferator‐activated receptor gamma coactivator 1‐alpha (*PGC‐1α*), glucose transporter 4 (*GLUT4*), and hexokinase II (*HKII*) genes. Fusion index analysis evaluated high‐glucose effects on myogenesis. RT‐qPCR examined spexin's effects on insulin receptor substrate 1 (*IRS‐1*), phosphatidylinositol 3‐kinase (*PI3K*), and *GLUT4* expression over different time points. Spexin concentrations at 1000 nM were not cytotoxic. High‐glucose incubation did not affect myogenesis yet was associated with downregulation of *PGC‐1α* and *GLUT4* genes, consistent with a transcriptional profile typically observed in insulin‐resistant states. Spexin‐treated C2C12 cells statistically significantly downregulated the expression of *IRS‐1* after 2 h of incubation. The effects of spexin on *IRS‐1*, *PI3K*, and *GLUT4* gene expression were comparable to metformin‐treated in insulin resistance C2C12 myotubes.

## Graphical abstract

This study investigated spexin in high‐glucose–induced insulin‐resistant C2C12 myotubes, focusing on its ability to modulate insulin‐signaling pathways and the glucose transporter GLUT4. These findings highlight spexin's potential role in improving peripheral insulin sensitivity by regulating key molecular targets involved in glucose homeostasis.

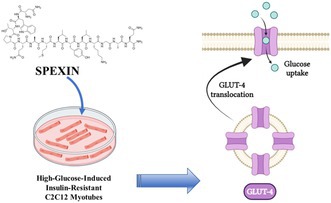

**Abbreviations**
AMPK: AMP‐Activated Protein Kinase
ANOVA: Analysis of Variance
ATCC: American Type Culture Collection
BSA: Bovine Serum Albumin
cDNA: Complementary DNA
DMEM: Dulbecco's Modified Eagle Medium
DMSO: Dimethyl Sulfoxide
ER: Endoplasmic Reticulum
FBS: Fetal Bovine Serum
GALR2/3: Galanin Receptor 2/3
GAPDH: Glyceraldehyde‐3‐Phosphate Dehydrogenase
GLUT4: Glucose Transporter Type 4
HKII: Hexokinase II
HS: Horse Serum
IRS‐1: Insulin Receptor Substrate 1
MAPK: Mitogen‐Activated Protein Kinase
MRF4: Myogenic Regulatory Factor 4
MTS: 3‐(4, 5‐Dimethylthiazol‐2‐yl)‐5‐(3‐carboxymethoxyphenyl)‐2‐(4‐sulfophenyl)‐2H‐tetrazolium
Myf5: Myogenic Factor 5
MyHC: Myosin Heavy Chain
PBS: Phosphate‐Buffered Saline
PCR: Polymerase Chain Reaction
PGC‐1α: Peroxisome Proliferator‐Activated Receptor Gamma Coactivator 1‐Alpha
PI3K: Phosphoinositide 3‐Kinase
PMS: Phenazine Methosulfate
RT‐qPCR: Quantitative Reverse Transcriptase PCR
SEM: Standard Error of the Mean
T1DM: Type 1 Diabetes Mellitus
T2DM: Type 2 Diabetes Mellitus
TBE: Tris‐Borate‐EDTA
TNFα: Tumor Necrosis Factor Alpha

## 1 Introduction

Diabetes mellitus is a metabolic disorder which manifests as hyperglycaemia, a pathological condition depicted by persistently elevated blood glucose levels [1]. Diabetes mellitus can be classified into several categories: type 1 diabetes mellitus (T1DM), type 2 diabetes mellitus (T2DM), gestational diabetes mellitus and specific cause types, including monogenic diabetes syndromes and chemical or drug‐induced diabetes [2]. According to the International Diabetes Federation, the global prevalence of diabetes is predominantly T2DM. The number of T2DM cases was estimated as 536.6 million in 2021 but may rise to 783.2 million in 2045 [3]. T2DM is a multifactorial disease with associated risk factors such as age, genetic predisposition, high‐fat diets, obesity and a sedentary lifestyle. Furthermore, T2DM often involves progressive loss of insulin secretion leading to relative insulin deficiency and is commonly associated with hyperglycaemia, consequent to insulin resistance, pancreatic beta cell dysfunction or both [2].

Insulin resistance occurs when peripheral tissues, namely the adipose tissue, liver and muscle exhibit reduced insulin sensitivity and responsiveness despite elevated insulin levels [4]. Chronic exposure to high insulin levels may desensitize insulin signaling pathways in peripheral tissues by ablating the expression of insulin receptors, and degradation of insulin receptor substrate 1 (*IRS‐1*) and impeding the phosphorylation of *IRS‐1* impairs the signal transduction [5]. As T2DM progresses, increased insulin production fails to compensate for diminished insulin sensitivity [1]. Meanwhile, chronic hyperinsulinaemia induces beta‐cell dysfunction and apoptosis through endoplasmic reticulum stress‐mediated and mitochondrial apoptotic pathways [6]. Hence, insulin deficiency could be due to reduced beta‐cell mass and function combined with hyperinsulinaemia‐induced insulin desensitization. Ultimately, this leads to impaired glucose uptake and contributes to insulin resistance and hyperglycaemia.

Conventional T2DM treatments target glucose regulation by enhancing insulin sensitivity through stimulation of insulin secretion and reduction of carbohydrate absorption [7]. However, long‐term administration of these drugs often leads to undesired side effects, including gastrointestinal discomfort, hypoglycaemia, or increased risk of diabetic ketoacidosis [8]. Additionally, GLP‐1 receptor agonists, which require frequent injections, can be inconvenient and reduce patient adherence [9]. Given the projected rise in T2DM prevalence, there is an increasing demand for alternative therapeutic approaches that offer reduced side effects while providing therapeutic effectiveness. Recently, peptides have garnered attention as suitable candidates for the current antidiabetic agents due to their prominent role in cellular metabolism regulation, high potency and selectivity, as well as low toxicity [10]. Among these peptides, spexin has emerged as a promising candidate due to its involvement in energy metabolism and glucose homeostasis [11]. As a functional agonist of galanin receptors 2 and 3 (GALR2 and GALR3), spexin plays a crucial role in regulating insulin sensitivity and glucose metabolism [12].

Spexin, also known as neuropeptide Q, is a peptide hormone discovered through the bioinformatics analysis of the human proteome and various species genomes [13, 14]. Spexin has ubiquitous expression in central and peripheral tissues, including the brain, gastrointestinal tract, gonads, liver, and skeletal muscles [14]. Various clinical studies reported lower circulating spexin in adults with T2DM compared with healthy controls [15, 16, 17, 18, 19, 20]. In these studies, reduced spexin has frequently been associated with higher fasting blood glucose, glycated hemoglobin (HbA1c), and homeostatic model assessment of insulin resistance (HOMA‐IR), suggesting that low spexin levels are associated with insulin resistance and impaired glucose metabolism [16, 20, 21, 22]. However, the low spexin levels and glycaemic indices could be improved through a 6 month lifestyle intervention program in prediabetic patients, further signifying their association [23]. While the consensus suggests a lower serum spexin level in T2DM patients, contradicting findings have been reported. Studies in adolescents and Iranian adults reported similar spexin levels in T2DM and non‐diabetic groups [24, 25] suggesting age and population differences may influence this relationship.

In animal models, exogenous spexin improves indices of glucose homeostasis and insulin sensitivity. Studies showed that spexin exerts hypoglycaemic effects in high‐fructose and high‐fat/streptozocin‐induced rodent models, improves insulin action and tolerance, and enhances pancreatic islet viability [26, 27, 28, 29]. Previous studies showed that in Sprague–Dawley rats with HFD‐induced insulin resistance and in HepG2 cells/primary mouse hepatocytes rendered insulin resistant, spexin lowers HOMA‐IR, suppresses hepatic glucose production and downregulates the gluconeogenic enzymes PEPCK and G‐6‐Pase via inhibition of the FoxO1/PGC‐1α pathway [17]. Meanwhile, spexin reduces HOMA‐IR and shows hypoglycaemic, hyperinsulinaemic and anti‐inflammatory actions via activation of PPARγ and AMPK in Albino Wistar rats with high‐fructose diet–induced metabolic syndrome (Said et al., 2023 [27]).

Despite growing evidence that spexin improves glycaemic control, there is limited study on its mechanism in improving insulin sensitivity, especially in skeletal muscle. The role of skeletal muscle in this process, being the primary site of insulin‐stimulated glucose disposal, remains incompletely understood. This gap is particularly important as the effects of spexin on key insulin‐signaling components such as insulin receptor substrate‐1 (IRS‐1), phosphoinositide 3‐kinase (PI3K), and glucose transporter type 4 (GLUT‐4) in skeletal muscle remain unclear. Skeletal muscle accounts for most postprandial glucose uptake via GLUT‐4, which is regulated by insulin‐dependent IRS‐1/PI3K/Akt signaling and insulin‐independent AMPK/p38 MAPK pathways [30, 31, 32]. Findings revealed that spexin activates GALR2, increases p38 MAPK and Akt phosphorylation, enhances glucose uptake in L6 myotubes, and improves glucose and insulin tolerance in vivo, but it remains unclear whether spexin can reverse the downregulation of key insulin‐signaling genes (IRS‐1, PI3K, GLUT‐4) characteristic of insulin‐resistant states [33, 34, 35, 36]. Hence, this study will elucidate the effects of spexin on IRS‐1, PI3K, and GLUT‐4 expression in an in vitro skeletal muscle under high glucose conditions, which aims to explore the potential of spexin relevance to enhance insulin‐stimulated glucose uptake and the possible pharmacological application of spexin in T2DM management.

## 2 Materials and Methods

### 2.1 Cell Culture

Murine C2C12 myoblast cells (CRL‐1772) were purchased from the American Type Culture Collection (ATCC, USA) (RRID:CVCL_0188). C2C12 myoblasts were cultured and maintained in a growth medium comprising Dulbecco's Modification of Eagle Medium (DMEM 1X with 25 mM glucose, Corning, USA) supplemented with 10% fetal bovine serum (FBS, BioSera, South America). The cells were maintained in a humidified incubator at 37°C with 5% CO2. The cells were routinely passaged at subconfluence (50%–65%) to prevent premature fusion and maintain the myoblastic population.

### 2.2 C2C12 Cells Differentiation Assay

Briefly, C2C12 myoblasts were seeded and cultured in DMEM containing 10% FBS until the cells reached 100% confluence. Upon confluency, the growth medium was replaced with a differentiation medium constituting DMEM supplemented with 2% horse serum (HS, Gibco, New Zealand) to induce myogenesis. The differentiation duration was 6 days, with the media refreshed every 2 days (Appendix A).

### 2.3 Hematoxylin and Eosin Staining

The fusion index was evaluated to determine the successful differentiation of C2C12 myoblasts into myotubes. Hematoxylin and eosin (H&E) staining was used to observe morphological changes associated with myotube formation. Firstly, a total of 5 × 10^4^ cells/well C2C12 myoblasts were seeded in a 24‐well plate and differentiated as described above in Section 2.2. On days 0, 1, 3 and 6, the cells were fixed with ice‐cold absolute ethanol (Fisher Bioreagents, Belgium) at −20°C for 7 min. Hematoxylin 560 MX (Leica Biosystems Richmond Inc., USA) was applied as a nuclear stain for 5 min, followed by three washes with Milli‐Q water. Next, Eosin 515 LT (Leica Biosystems Richmond Inc., USA) was added for 30 s, and the cells were again washed three times with Milli‐Q water. After an additional rinsing step with phosphate‐buffered saline (1× PBS, Biomedia, Singapore), the cells were allowed to air‐dry at room temperature. For each experimental condition, images were obtained from five randomly selected fields per well, from three independent experiments. Image acquisition and subsequent qualitative assessment were performed by an investigator blinded to group allocation during both imaging and analysis. The images were captured with a microscope (Olympus CKX41SF, Japan) equipped with a camera (Olympus DP21, Japan). In each image, the fusion index was analyzed using ImageJ software (National Institutes of Health, US). The threshold number of myonuclei for a cell to be considered a myotube was arbitrarily set as three. The fusion index is defined as the ratio of myonuclei present in myotubes to the total nuclei in the image and was calculated with the following formula:

$$\text{Fusion index}\;\left(\%\right)=\frac{\text{Number of nuclei in cells with}\geq 3\;\text{nuclei}}{\text{Total nuclei present in}\;a\;\text{given field}}\times 100\%$$

### 2.4 Spexin and Metformin Preparation

Human spexin was synthesized by Genscript, Singapore (sequence: NWTPQAMLYLKGAQ, 97.4% purity) and metformin hydrochloride was purchased from Sigma‐Aldrich, USA. Different concentrations of lyophilized spexin and metformin powder were dissolved in autoclaved Milli‐Q water and filtered with a 0.22‐μm pore size polyethersulfone (PES) membrane sterile filter (Bioflow Lifescience Sdn Bhd, Malaysia). Subsequently, the solutions were diluted in DMEM supplemented with 0.2% bovine serum albumin (BSA) (Sigma‐Aldrich, USA) to obtain the specific working concentrations.

### 2.5 Cell Viability Assay

Cell proliferation was assessed using the ability of the cells to reduce 3‐(4, 5‐dimethylthiazol‐2‐yl)‐5‐(3‐carboxymethoxyphenyl)‐2‐(4‐sulfophenyl)‐2H‐tetrazolium (MTS) to form readily soluble purple‐brown formazan products. The MTS stock solutions were prepared according to the manufacturer's instructions, comprising 2.0 mg/mL CellTiter 96 AQueous MTS reagent powder (Promega, USA) and 0.21 mg/mL reducing agent phenazine methosulfate (PMS, MP Biomedicals, USA). Briefly, C2C12 myoblasts were seeded at a seeding density of 10^4^ cells/well in a 96‐well plate. Upon differentiation, the C2C12 myotubes were treated with increasing spexin concentrations (100 nM, 200 nM, 400 nM, 800 nM, and 1000 nM) for 24 h. Two control groups consisting of negative control (without spexin) and positive control (10% dimethyl sulfoxide; DMSO, Sigma‐Aldrich, USA) were included in this study. After the 24 h treatment, 20 μL MTS reagent was directly added to the wells and the plate was incubated for 2 h. The absorbance readings were measured at 492 nm on a Tecan SUNRISETM microplate reader (Tecan Austria GmbH, Austria) equipped with Magellan v7.2 software. Cell viability was reported as a percentage relative to the normal control using the calculation as shown below:

$$\text{Cell viability}\;\left(\%\text{relative to normal control}\right)=\frac{\text{Sample absorbance}\;\left(492\;\mathrm{nm}\right)}{\text{Normal control absorbance}\;\left(492\;\mathrm{nm}\right)}\times 100\%$$

### 2.6 Induction of Insulin Resistance Model Using High Glucose Exposure

High glucose mimics a state of hyperglycaemia, as in the primary pathophysiology of T2DM. Therefore, insulin‐resistant C2C12 myotubes were established with high glucose, as described previously [37]. It was reported that the glucose concentration of 60 mM could induce insulin resistance but did not exert cytotoxicity in differentiating C2C12 myoblasts [37].

Briefly, D‐glucose (Millipore Sigma, France) was dissolved in DMEM at room temperature and filtered to prepare a 60 mM high‐glucose‐DMEM working stock. Insulin resistance was induced by incubating C2C12 myoblasts in a high‐glucose differentiation medium (60 mM‐glucose DMEM supplemented with 2% horse serum) for 6 days. The ATCC recommends a glucose concentration of 25 mM for routine cell culture maintenance. Therefore, this glucose concentration was considered the normal or basal growth condition. The effects of high glucose incubation on myogenesis were assessed using H&E staining and fusion index calculation, as outlined in Section 2.3.

### 2.7 Total RNA Extraction and cDNA Conversion

Total RNA was extracted using Quick‐RNA Miniprep Kit (Zymo Research, USA) with an on‐column DNase I digestion according to the manufacturer's protocol. RNA concentration and purity (A260/A280) were assessed using NanoDrop One (Thermo Scientific, USA). Subsequently, a high‐capacity cDNA reverse transcription kit (Applied Biosystems, US) was used to convert the total extracted RNA into single‐stranded complementary DNA (cDNA) based on the manufacturer's protocol.

### 2.8 Gene Expression Analysis Using Polymerase Chain Reaction (PCR)

Gene expression analysis of undifferentiated myoblasts and differentiated myotubes was determined using polymerase chain reaction (PCR). PCR amplification was performed using PCRBIO HS Taq Mix (PCR Biosystems, UK) in the SimpliAmp thermal cycler (Thermo Scientific, USA). The final volume of the PCR mix was 25 μL, comprising 200 nM primers and 20 ng cDNA. The PCR cycling conditions were as follows: 3 min of initial denaturation at 95°C, followed by 35 cycles of denaturation (95°C, 30 s), annealing (58°C, 30 s) and extension (72°C, 1 min) with a final extension step of 10 min at 72°C.

The electrophoresis of the amplified PCR products was performed on a 2% agarose gel (Vivantis Inc., USA) in 0.5× TBE buffer (Tris‐borate‐EDTA buffer, Sigma, USA). The gel was run at 100 V for 40 min using ADVANCE Mupid‐One (Takara, Japan). Bands were visualized using the Azure 300 imaging system (Azure Biosystems, USA). Primer sequences used for the expression analysis of various genes are detailed in Table 1.

**TABLE 1:** Primer sequences used in PCR to confirm the differentiation of C2C12 myoblasts into myotubes.

Gene	Primer sequence (5 to 3)	Amplicon size (bp)
*Myf5*	F: CACCACCAACCCTAACCAGAG R: AGGCTGTAATAGTTCTCCACCTG	114
*MyHC*	F: ACCGAAGGCGGAACTACTGTAAC R: ATCCAGGCTGCATAACGCTCT	152
*MRF4*	F: ATTCTTGAGGGTGCGGATTTC R: CCTTAGCAGTTATCACGAGGC	91
*GAPDH*	F: GGAGAGTGTTTCCTCGTCCC R: ATGAAGGGGTCGTTGATGGC	136

Abbreviations: F, forward primer; *GAPDH*, glyceraldehyde‐3‐phosphate dehydrogenase; *MRF4*, myogenic regulatory factor 4; *Myf5*, myogenic factor 5; *MyHC*, myosin heavy chain; R, reverse primer.

### 2.9 Gene Expression Analysis Using Quantitative Reverse Transcriptase PCR (RT‐qPCR)

For the characterization of the high‐glucose conditions in C2C12 model at the transcriptional level, C2C12 myoblasts were differentiated either in normal glucose (25 mM) or high glucose (60 mM) differentiation media. The expression levels of genes previously associated with insulin resistance, including *PGC‐1α*, *GLUT4*, and *HKII*, were compared between these two groups.

Meanwhile, the effects of spexin were evaluated by exposing C2C12 myotubes cultured under high‐glucose conditions to three different conditions: untreated cells serving as the negative control, cells treated with spexin as the experimental group and cells exposed to 1 mM metformin as the positive control. The treatments were applied for 1, 2, 4, and 24 h to assess the time‐dependent effects. The treatment medium used was DMEM supplemented with 0.2% BSA. The expression levels of key mediators including *IRS‐1*, *PI3K*, and *GLUT4* were analyzed across the different groups. Metformin, a gold standard for T2DM treatment, served as the positive control at a concentration of 1 mM, which was shown to be non‐cytotoxic to differentiated C2C12 myotubes following 48 h of incubation. The primer sequences of target genes used for characterizing the high‐glucose model and assessing the effects of spexin on insulin resistance–related genes of C2C12 myotubes were included in (Table 2 and Table 3), respectively.

**TABLE 2:** Primer sequences used in RT‐qPCR to assess expression of insulin resistance–related genes in C2C12 myotubes.

Gene	Primer sequence (5 to 3)
*PGC‐1α*	F: AACCAGTACAACAATGAGCCTG R: AATGAGGGCAATCCGTCTTCA
*GLUT4*	F: TCCTTCTATTTGCCGTCCTC R: GGTTTCACCTCCTGCTCTAA
*HKII*	F: GGAGGAGGAGCAGTATGG R: TTCAGCCGTGTGAGGTAA
*GAPDH*	F: GGCACAGTCAAGGCTGAGAATG R: ATGGTGGTGAAGACGCCAGTA

Abbreviations: F, forward primer; *GAPDH*, glyceraldehyde‐3‐phosphate dehydrogenase; *GLUT4*, glucose transporter type 4; *HKII*, hexokinase II; *PGC‐1α*, peroxisome proliferator‐activated receptor gamma coactivator 1‐alpha; R, reverse primer.

**TABLE 3:** Primer sequences used in RT‐qPCR to study the effects of spexin on the expression of insulin‐signaling–related genes in insulin‐resistant C2C12 myotubes.

Gene	Primer sequence (5‐3)
*IRS‐1*	F: ACGAACACTTTGCCATTGCC R: CCTTTGCCCGATTATGCAGC
*PI3K*	F: AAGCCATTGAGAAGAAAGGACTG R: ATTTGGTAAGTCGGCGAGATAG
*GLUT4*	F: TCCTTCTATTTGCCGTCCTC R: GGTTTCACCTCCTGCTCTAA
*GAPDH*	F: GGCACAGTCAAGGCTGAGAATG R: ATGGTGGTGAAGACGCCAGTA

Abbreviations: F, forward primer; *GAPDH*, glyceraldehyde‐3‐phosphate dehydrogenase; *GLUT4*, glucose transporter type 4; *IRS‐1*, insulin receptor substrate 1; *PI3K*, phosphoinositide 3‐kinase; R, reverse primer.

Briefly, the C2C12 myoblasts were seeded in a 24‐well plate at a seeding density of 5 × 10^4^ cells/well and differentiated as described above (Section 2.2), either in normal or high glucose concentrations. Following RNA extraction and conversion to cDNA (Section 2.7), amplification was carried out using qPCRBIO SyGreen Blue Mix (PCR Biosystems, UK) according to the manufacturer's protocol, utilizing the CFX Duet Real‐Time PCR System (Bio‐Rad, USA). The PCR cycling conditions were as follows: 2 min of polymerase activation at 95°C, followed by 40 cycles of denaturation (95°C, 5 s) and annealing/extension (60°C, 30 s).

Melt curve analysis was performed using the housekeeping gene, glyceraldehyde‐3‐phosphate dehydrogenase (*GAPDH*) to normalize against the expression of the gene of interest. The relative expression was calculated using the comparative Ct method with the equation below and expressed as the fold change relative to the untreated control.

$$\text{Relative expression}=2^{-\text{ΔΔCt}},\text{where}$$

$$\text{ΔΔCt}=\left[{\left(\mathrm{Ct}\;\text{target gene}–\mathrm{Ct}\;\text{reference gene}\right)}_{\text{treated}}–{\left(\mathrm{Ct}\;\text{target gene}–\mathrm{Ct}\;\text{reference gene}\right)}_{\text{untreated}}\right]$$

### 2.10 Statistical Analysis

All experiments were performed with three biological replicates unless specified otherwise. In this study, biological replicates correspond to independent experiments performed with separate cell seeding, differentiation, treatments, and RNA extraction. Within each biological replicate, technical replicates were obtained from three wells per condition; for each condition, technical replicates were averaged to yield a single value per biological replicate.

Results were expressed as mean ± standard error of the mean (SEM). For comparison between two treatment groups, data were analyzed using a two‐tailed unpaired Student's *t*‐test; for comparison among three or more treatment groups, one‐way analysis of variance (ANOVA) was used, with a follow‐up Tukey's post hoc test where necessary. Statistical analyses were carried out using GraphPad Prism v8.0.2 (GraphPad Software, San Diego, California USA). Results with *p*‐values ≤ 0.05 were considered statistically significant.

## 3 Results

### 3.1 Differentiation of C2C12 Myoblasts Into Myotubes

**FIGURE 1:**
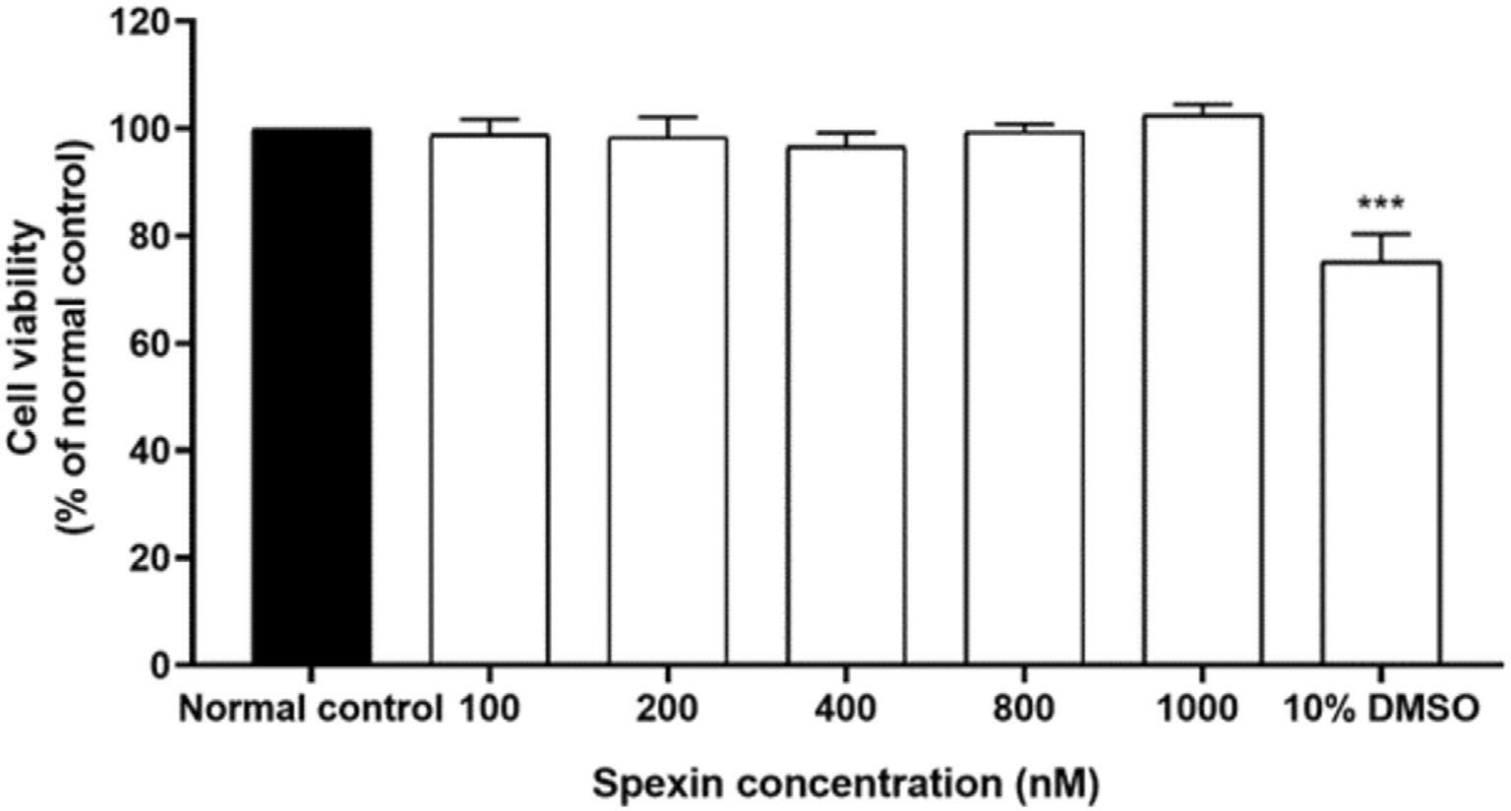
Effects of increasing concentrations of spexin (in nM) on the cell viability of C2C12 myotubes after 24 h of treatment as determined by MTS assay. Two controls were used including negative control group (untreated) and positive control group (10% DMSO). The values were represented as mean ± SEM (*n* = 3). Significance compared to the normal control group (black bar) was evaluated with a one‐way ANOVA test, with a follow‐up Tukey test. ***, *p* < 0.005.

C2C12 myoblasts were successfully differentiated into myotubes, as evidenced by the formation of multinucleated myotube‐like structures confirmed through H&E staining and gene expression analysis, respectively (Appendices B and C). Both the undifferentiated myoblasts and differentiated cells at days 1, 3, and 6 were stained with H&E to determine the fusion index in order to assess the progression of C2C12 differentiation. It was shown that the number of myotubes progressively increased from day 0 to day 6 (Appendix B i, ii, iii, and iv) with myoblasts observed across the differentiation duration from day 0 to day 6. As shown in Figure 1E, there was a significant time‐dependent increase in the fusion index of differentiating C2C12 cells, from 0% on day 0 to 14.32% on day 3 to 24.76% on day 6 (*p* < 0.005). There was no statistically significant increase in the fusion index percentage as observed on day 1 (Figure 1E). Since day 6 exhibited the highest fusion index of myotube formation, the differentiation duration was set as 6 days for subsequent experiments.

Meanwhile, the expression of differentiation markers of *Myf5*, *MyHC* and *MRF4* were compared between undifferentiated myoblasts and differentiated myotubes to validate the results from H&E staining. A distinct band was present in C2C12 cells for *Myf5* before and after differentiation as shown in Appendix C For *MyHC*, while bands were present for both myoblasts and myotubes, there was an obvious higher band intensity after 6 days of differentiation. For *MRF4*, a faint band was present in differentiated myotubes on day 6 of differentiation but absent in myoblasts. The consistent intensity of the GAPDH gel band between myoblasts and myotubes were shown in this experiment.

### 3.2 Cytotoxicity Effects of Different Concentration of Spexin

MTS assay assessed the potential cytotoxic effects of spexin on C2C12 myotubes after a 24 h treatment as shown in Figure 1. The spexin concentrations ranging from 100 to 1000 nM were prepared in this study. Spexin did not significantly affect cell viability across the tested concentrations. Since spexin did not exhibit cytotoxicity up to 1000 nM with cell viability of 102.55% (*p* > 0.05), the spexin concentration at 1000 nM was used for subsequent experiments. Meanwhile, the 10% DMSO concentration was used as a standard solvent without significant cytotoxicity.

### 3.3 Effects of High Glucose on Differentiation in C2C12 Myotubes

C2C12 myotubes were incubated in a high glucose differentiation medium containing 60 mM glucose for 6 days to induce insulin resistance in the myotubes. C2C12 cells differentiated in media containing either normal glucose concentration (25 mM) or high glucose concentration (60 mM) were stained with H&E. The fusion index was determined to assess the effects of high glucose on myogenesis.

**FIGURE 2:**
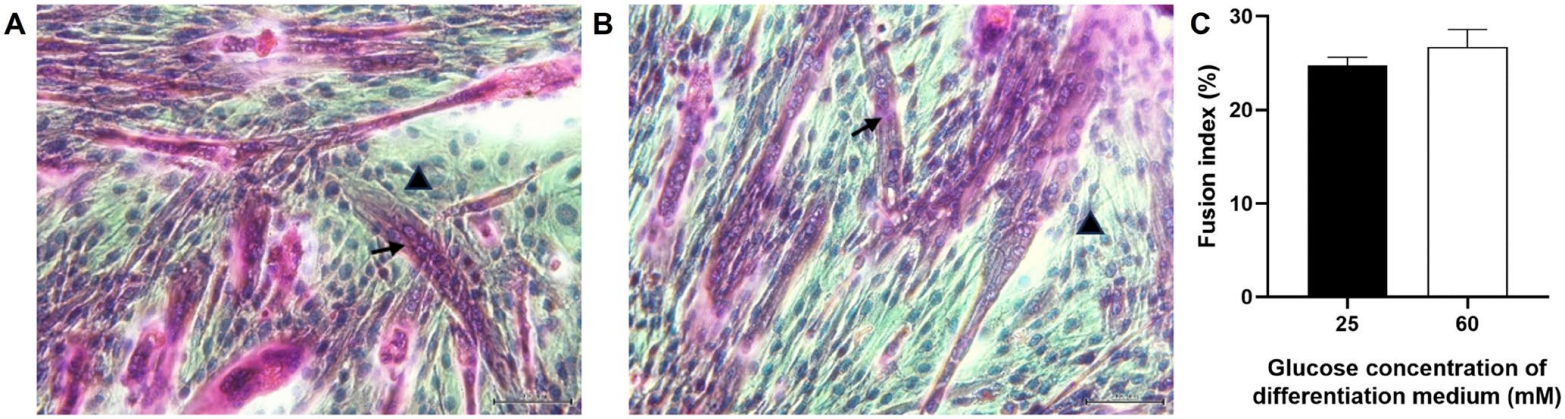
Effects of different glucose concentrations in differentiation medium on C2C12 cell differentiation as assessed by H&E staining and fusion index quantification. Bright‐field images of H&E‐stained cells were taken under the same magnification (x200) for differentiated C2C12 myotubes in (A) normal glucose concentration (25 mM) and (B) high glucose concentration (60 mM) differentiation media. The arrows indicate the multinucleated myotubes whereas the arrowheads indicate the unfused myoblasts. Scale bar, 200 μm. (C) Quantification of fusion index (in %) in C2C12 cells upon 6 days of differentiation in normal (25 mM) and high (60 mM) glucose differentiation media. The values were represented as mean ± SEM (*n* = 3). Significance compared to the 25 mM group (black bar) was evaluated by a two‐tailed unpaired Student's *t*‐test.

There were no apparent morphological differences in myotube formation between the two glucose concentrations (Figure 2A,B). Although the fusion index for C2C12 myotubes differentiated in the high‐glucose medium was slightly higher (26.69%) as compared to the normal‐glucose medium (24.76%), the effects were not significant (Figure 2C). Since high glucose treatment did not interfere with the initial differentiation process, C2C12 cells were incubated directly in high glucose differentiation (60 nM) media upon 100% confluence to induce cellular insulin resistance.

### 3.4 Gene Expression of Insulin Resistance–Related Markers

**FIGURE 3:**
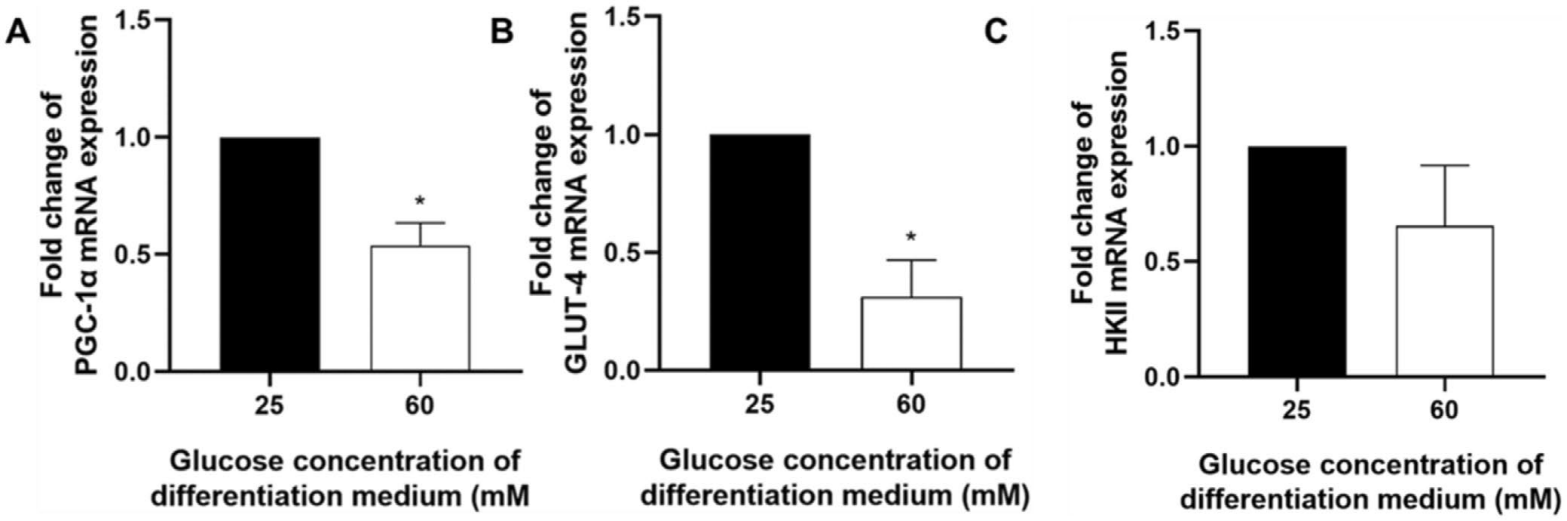
Fold change in mRNA expression of normoglycaemic and hyperglycaemic C2C12 myotubes as assessed by RT‐qPCR for insulin resistance markers: (A) *PGC‐1α*; (B) *GLUT4*; (C) *HKII*. The cells were incubated in differentiation media containing either normal (25 mM) or high (60 mM) glucose concentrations for 6 days. The housekeeping gene used was GAPDH. The fold change in mRNA expression was relative to the control group (25 mM glucose, black bar). The values were represented as mean ± SEM (*n* = 3). The data were analyzed with a two‐tailed unpaired Student's t‐test. *, *p* < 0.05.

The expression levels of three insulin resistance biomarkers including *PGC‐1α*, *GLUT4*, and *HKII* were quantified using RT‐qPCR and compared between cells incubated in normal‐glucose (25 mM) and high‐glucose (60 mM) differentiation media. In high‐glucose‐differentiated C2C12 myotubes, the gene expression of *PGC‐1α* was statistically significantly lower than in normal glucose concentration, indicating changes in insulin‐pathway–related gene expression consistent with an insulin‐resistant–like state (Figure 3A,B). Meanwhile, the expression levels of *GLUT4* and *HKII* were slightly lower in high glucose concentration (60 mM) as compared to normal glucose concentration, as shown in Figure 3C.

### 3.5 Gene Expression of Analysis of Key Insulin‐Dependent Pathway Genes at Different Time Points

**FIGURE 4:**
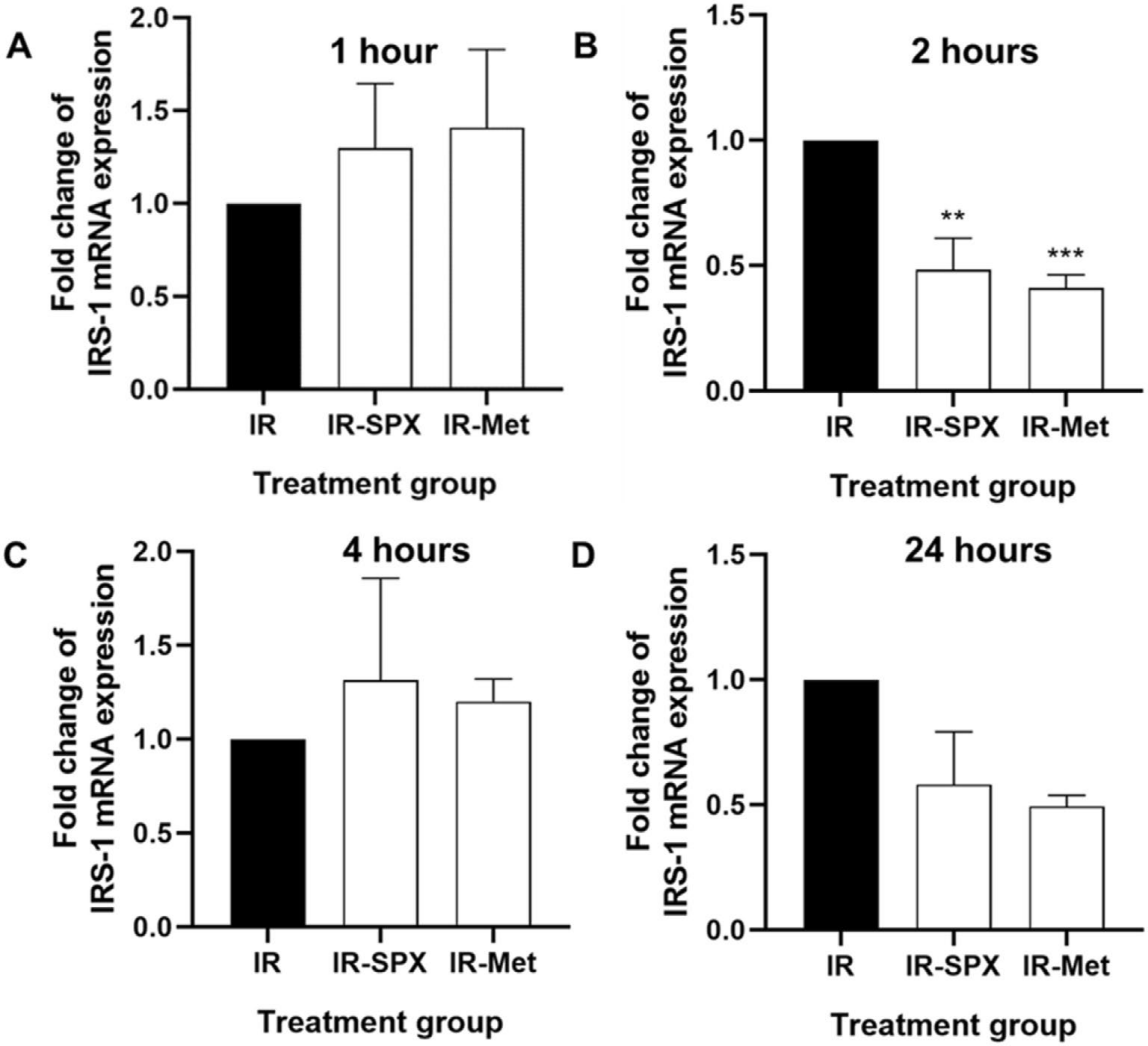
Fold change in mRNA expression of *IRS‐1* in insulin‐resistant C2C12 myotubes as assessed by RT‐qPCR at different time points: (A) 1 h; (B) 2 h; (C) 4 h; (D) 24 h. Insulin‐resistant cells were treated with 1000 nM spexin (experimental group, IR‐SPX) or 1 mM metformin (positive control, IR‐Met), and the fold change in mRNA expression was relative to the control group (untreated control, IR; black bar). The housekeeping gene used was GAPDH. The values were represented as mean ± SEM (*n* = 3). The data were analyzed with one‐way ANOVA with a follow‐up Tukey test, performed only when the ANOVA test was significant. **, *p* < 0.01; ***, *p* < 0.005.

RT‐qPCR was used to assess the effects of spexin on three key regulatory effectors of the insulin‐dependent pathway: *IRS‐1*, *PI3K*, and *GLUT4*. The changes in gene expression levels of the experimental (1000 nM spexin) and positive control (1 mM metformin) groups were compared to untreated insulin‐resistant C2C12 myotubes. Spexin significantly reduced the expression of *IRS‐1* after 2 h of treatment (*p* < 0.01) (Figure 4B). Meanwhile, the upregulation of *IRS‐1* at 1 h and 4 h, as well as its downregulation at 24 h upon spexin treatment, were exhibited in Figure 4A,C,D, respectively.

The regulation of *IRS‐1* expression following metformin treatment at 1, 2, 4 and 24 h, exhibited a pattern consistent with that observed during spexin treatment relative to the untreated control group as shown in Figure 4. Overall, the fold change in *IRS‐1* expression varied inconsistently across the time points for both treatments.

**FIGURE 5:**
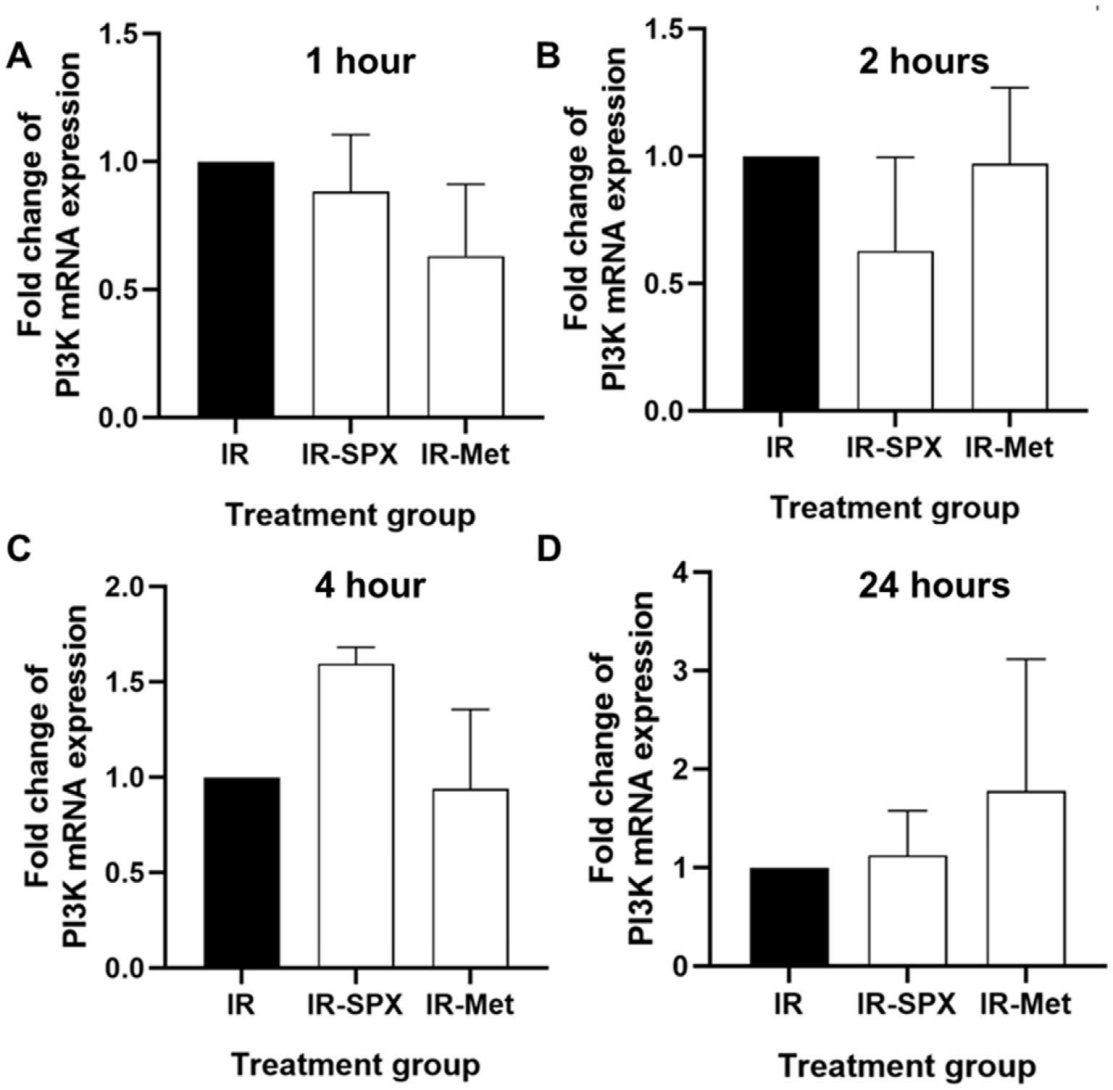
Fold change in mRNA expression of *PI3K* in insulin‐resistant C2C12 myotubes as assessed by RT‐qPCR at different time points:(A) 1 h; (B) 2 h; (C) 4 h; (D) 24 h. Insulin‐resistant cells were treated with 1000 nM spexin (experimental group, IR‐SPX) or 1 mM metformin (positive control, IR‐Met), and the fold change in mRNA expression was relative to the control group (untreated control, IR; black bar). The housekeeping gene used was GAPDH. The values were represented as mean ± SEM (*n* = 3). The data were analyzed with one‐way ANOVA.

For *PI3K* expression levels, there were no statistically significant observations in both the spexin and metformin‐treated groups following 1, 2, 4 and 24 h of incubation as shown in Figure 5. Upon spexin treatment, the *PI3K* expression level was downregulated at 1 and 2 h but remained upregulated at 4 and 24 h as compared to the untreated group. A time‐dependent effect was not apparent as the fold change of *PI3K* expression level peaked after 4 h of spexin treatment. In contrast, metformin treatment showed that *PI3K* expression was downregulated at the first hour and upregulated at 24 h with almost no changes at 2 h and 4 h of incubation.

**FIGURE 6:**
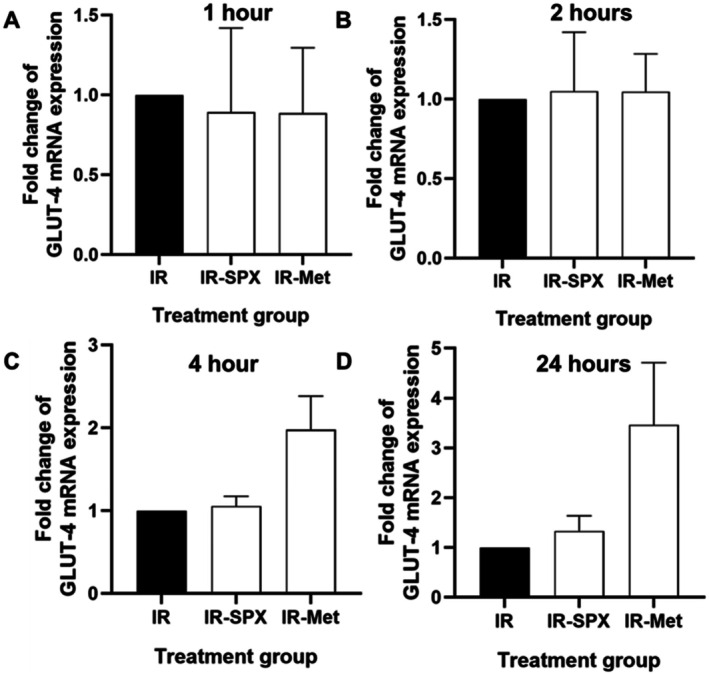
Fold change in mRNA expression of GLUT4 in insulin‐resistant C2C12 myotubes as assessed by RT‐qPCR at different time points: (A) 1 h; (B) 2 h; (C) 4 h; (D) 24 h. Insulin‐resistant cells were treated with 1000 nM spexin (experimental group, IR‐SPX) or 1 mM metformin (positive control, IR‐Met), and the fold change in mRNA expression was relative to the control group (untreated control, IR; black bar). The housekeeping gene used was GAPDH. The values were represented as mean ± SEM (*n* = 3). The data were analyzed with one‐way ANOVA.

As shown in Figure 6, spexin and metformin treatments did not result in significant changes in *GLUT4* gene expression levels after 1, 2, 4, and 24 h. Upon spexin treatment, the *GLUT4* expression level was downregulated at 1 h but remained upregulated at 2 h, 4 h, and 24 h as compared to the untreated group. A time‐dependent effect was evident, with the fold change in *GLUT4* mRNA expression peaking at 24 h post‐treatment, though the changes were not statistically significant. Similarly, metformin treatment exhibited a time‐dependent trend: *GLUT4* expression was downregulated at 1 h and upregulated at 2, 4, and 24 h, but these effects were also not statistically significant.

## 4 Discussion

Spexin has demonstrated significant hypoglycaemic effects in animal models, improving insulin sensitivity, tolerance, and pancreatic islet viability [27] [26]. Furthermore, reduced spexin expression in individuals with T2DM highlights its potential as a therapeutic target for managing insulin resistance [15]. Since skeletal muscle is responsible for approximately 80% of insulin‐stimulated glucose uptake, enhancing insulin responsiveness in this tissue is critical for glycaemic control in T2D. In this study, C2C12 myotubes were used as an in vitro skeletal muscle model to examine high‐glucose–induced, insulin‐resistant–like changes in insulin pathway–related signaling, with a particular focus on the PI3K/Akt signaling, the key mechanisms in T2DM. Furthermore, C2C12 myoblasts can differentiate into myotubes and express insulin‐responsive GLUT4, thereby providing a relevant model to study how spexin may influence glucose handling at the level of skeletal muscle [38].

In this study, the successful differentiation of C2C12 myoblasts was confirmed by assessing the fusion index [Appendix B (v)] and the gene expression levels of *Myf5*, *MRF4* and *MyHC* (Appendix C). Myogenesis is the process by which myoblasts differentiate into myotubes and subsequently fuse to form myofibers [39]. Transcriptional factors, also known as myogenic regulatory factors including *Myf5* are the early activator of myogenesis. The *Myf5* is expressed during the activation, propagation and early phases of myogenesis, playing a crucial role in committing myoblasts to terminal differentiation [40]. *MRF4*, a late differentiation factor modulates myoblast lineage commitment and myocyte differentiated‐state maintenance and development [41]. Meanwhile, *MyHC* is a key marker of myotube maturation, representing a late differentiation marker by the formation of sarcomeric structure of a mature skeletal muscle [42].

The findings demonstrated that after 6 days of differentiation, the fusion index, which is indicative of the level of differentiation from myoblasts into myotubes, was 24.76% (Figure 2). This value was similar to that reported by Tanimoto et al. (2023) [40], which reported the fusion index of 24% for the same differentiation period. The statistically significant increase in the fusion index indicates successful C2C12 differentiation. This observation was validated by higher band intensities of late myogenic markers, *MyHC* and *MRF4*, after differentiation as shown in Appendix 2C. Similarly, previous studies have demonstrated an increased expression of *MyHC* and *MRF4* upon differentiation [43, 44]. Pre‐differentiated myoblasts had a faint *MyHC* band, implying that the gene expression is relatively lower but not absent. Previous studies reported similar findings with RT‐qPCR analysis and Western blot detection, which suggested that myoblasts express basal levels of *MyHC* [45].

The expression of *Myf5* remained consistent before and after differentiation in this study in concordance with the findings from da Paixão and colleagues (2021) [46]. In contrast, another study reported the absence of *Myf5* expression following differentiation [45] The discrepancy in results may be attributed to variations in differentiation methods and the confluency at which differentiation was initiated [45]. The sustained *Myf5* expression after differentiation may indicate the preservation of a progenitor cell pool, as *Myf5* is expressed in reserve cells. It was suggested that *Myf5* mRNA is consistently expressed in both proliferative and reserve cells but is completely absent in differentiated myotubes [47]. In addition, the fusion index of under 100% further suggests that the extent of the differentiation process is not sufficiently efficient, rather it implies the establishment of a myoblasts‐myotubes co‐culture. Next, the cytotoxic effects of different concentrations of spexin on C2C12 myotubes were evaluated using an MTS assay (Figure 1).

Spexin treatment at concentrations up to 1000 nM for 24 h showed no significant cytotoxicity, consistent with previous findings indicating that 1 to 1000 nM spexin was non‐cytotoxic to C2C12 cells [48] and other metabolic cell models where 1000 nM spexin produced clear functional effects [49]. Meanwhile, it was reported that 1000 nM spexin significantly promoted cell viability in C2C12 myoblasts, yet this effect was not observed in myotubes [48]. This discrepancy likely arises from differences in cell stages, as myoblasts remain proliferative whereas myotubes, under low‐serum conditions, are terminally differentiated and in growth arrest. Consequently, the proliferative effects of spexin on myoblasts do not extend to myotubes [48]. Therefore, while spexin may exert a proliferative effect in myoblasts, the same effect would be negligible on the number of viable myotubes, which remain in a state of growth arrest.

This study used chronic high glucose exposure to induce insulin resistance in C2C12 myotubes and assess its impact on myogenesis. High glucose mimics a state of hyperglycaemia, as in the primary pathophysiology of T2DM. Therefore, insulin‐resistant C2C12 myotubes were established with high glucose, as described previously by Luo et al. (2019) [37]. Based on the same study, a glucose concentration of 60 mM could induce insulin resistance but did not exert cytotoxicity in differentiating C2C12 myoblasts [37]. In this study, fusion index quantification (Figure 2C) revealed that incubation with high glucose (60 mM) differentiation medium had no significant effect on myogenesis as compared to normal glucose conditions (25 mM). Meanwhile, Kato et al. (2022) reported that high glucose concentrations did not alter myotubes morphology or the expression of myogenic differentiation markers including *Myf5* [50]. In another study, it was observed that hyperglycaemia inhibited myogenesis, as indicated by decreased myotube number and size [37]. In contrast to earlier findings, it was demonstrated that hyperglycaemia accelerated myogenesis with increased myonuclei number at the initial myogenic stages, but led to structural defects, impaired contractile function and premature senescence in differentiated myotubes [51]. The effects of high glucose on C2C12 myogenesis remain inconclusive, with conflicting results across studies. Hence, further research is needed to clarify the molecular mechanisms underlying glucotoxicity in myogenesis.

The C2C12 myotubes were cultured in high‐glucose differentiation medium for 6 days, and RT‐qPCR analysis revealed statistically significant downregulation of *PGC‐1α* but not *GLUT4* and *HKII*, suggesting partial induction of insulin resistance as exhibited in Figure 3. The selection of *PGC‐1α*, *GLUT4* and *HKII* as biomarkers in this study arises from their well‐documented roles in glucose metabolism and their involvement in the pathophysiology of insulin resistance. *PGC‐1α*, a key regulator of mitochondrial biogenesis, also promotes glucose metabolism by enhancing *GLUT4* expression and translocation [52]. Hence, its diminished activity decreases glucose uptake and mitochondrial fatty acid oxidation, resulting in the accumulation of partially oxidized fatty acid intermediates that trigger insulin resistance [53]. This effect may explain the association between decreased *PGC‐1α* expression and the manifestation of insulin resistance in patients [54]. Hyperglycaemia has been known to induce *PGC‐1α* downregulation, possibly by enhancing DNA methylation at the promoter site to repress its expression [55]. However, insulin‐stimulated functional assays such as glucose uptake assays were not performed in this model; therefore, the degree of functional insulin resistance cannot be directly confirmed and should be clarified in future studies.

*GLUT4*, which is the primary insulin‐mediated glucose transporter in skeletal muscle, is essential for efficient glucose uptake. In T2DM patients, *GLUT4* expression is significantly reduced, resulting in impaired glucose metabolism [56]. On the other hand, it was reported that hyperglycaemia‐mediated reactive oxygen species (ROS) production initiates endoplasmic reticulum (ER) stress and activates the protein kinase RNA‐like endoplasmic reticulum kinase (PERK) pathway, resulting in the degradation of *GLUT4* protein and subsequently reducing glucose uptake [57]. In this study, RT‐qPCR analysis of C2C12 myotubes cultured in a high‐glucose differentiation medium revealed significant downregulation of *PGC‐1α* expression as shown in Figure 3. These findings are consistent with their critical roles in glucose metabolism and insulin resistance and align with previous studies demonstrating similar downregulation of these markers in vitro insulin‐resistant models [33, 58].

*HKII* is the predominant hexokinase isoform in skeletal muscle and plays a crucial role in catalyzing glucose phosphorylation, which is the first step in glycolysis. Its levels have been reported to be reduced in the T2DM group [59]. Reduced *HKII* levels impair glucose trapping and metabolism, contributing to insulin resistance. With low *HKII* levels, the cells have an impaired ability to trap and metabolize the unphosphorylated glucose, resulting in poor glucose oxidation and utilization and ultimately manifesting into insulin resistance [60]. It is postulated that the high‐glucose condition suppresses *HKII* transcription by downregulating its transcription factor proliferator‐activated receptor γ (*PPARγ*) [61]. However, *HKII* expression did not show significant changes in this study (Figure 5). The discrepancy in *HKII* expression results may be attributed to differences in the methods used to induce insulin resistance, such as the palmitic acid treatment reported in another study [58]. It was postulated that palmitic acid and high glucose may induce insulin resistance via different molecular mechanisms. The non‐significant decrease in *HKII* expression could also suggest that the method for establishing insulin resistance in this study may not be optimally efficient. Therefore, further optimization of experimental conditions, such as glucose concentration and incubation duration, are required.

In skeletal muscles, glucose uptake can occur via the insulin‐dependent or insulin‐independent pathway. The insulin‐dependent pathway is activated when insulin binds to its receptor and leads to the sequential activation of *IRS‐1* followed by *PI3K* and *Akt*. The insulin‐independent pathway is triggered by metabolic stimuli such as exercise and activates AMPK along with its downstream kinases including p38 MAPK [30]. Both pathways involve a series of activation steps, which ultimately promote *GLUT4* translocation to the cell membrane to facilitate glucose uptake in muscles [30]. Insulin resistance disrupts the insulin‐dependent pathway as exemplified by the significantly downregulated expression of *IRS‐1*, *PI3K*, and *GLUT4* in diabetic rodent models, suggesting a possible coordinate regulation [62]. Similarly, their expression levels were diminished in C2C12 cells cultured in high‐glucose media [63]. Hence, this study investigated the effects of spexin on IRS‐1, PI3K, and GLUT4 expression in high‐glucose conditions of C2C12 myotubes, in order to explore potential transcriptional modulation of insulin‐related signaling components.

The RT‐qPCR was used to assess the time‐dependent effects of spexin on the gene expression of these insulin‐sensitivity genes in insulin‐resistant C2C12 myotubes, with metformin serving as the control. However, the failure of metformin to significantly improve the expression of key genes such as IRS‐1, PI3‐K, and GLUT4 as compared to the insulin‐resistant group presents a limitation of this study as shown in Figure 4 to Figure 6. Although metformin is often considered the gold standard for improving insulin sensitivity, its suboptimal effect in this study could be attributed to several factors such as the treatment duration, concentration, or the experimental model used.

The *IRS‐1* expression with spexin treatment in C2C12 myotubes was statistically significantly downregulated after 2 h of spexin treatment, with fluctuations at different time points (Figure 4). The recovery of *IRS‐1* expression at 4 h suggests a compensatory feedback mechanism activated by acute treatment, potentially mediated by other regulatory molecules. Nevertheless, the reduced *IRS‐1* expression at 4 and 24 h did not necessarily correspond to the exacerbation of insulin resistance. A study from Chibalin and colleagues (2000) [64] reported a marked increase in *IRS‐1* tyrosine phosphorylation and *IRS‐1*‐associated *PI3K* activity upon insulin stimulation of rat muscles, despite the reduced *IRS‐1* expression. These findings suggest that the improved insulin responsiveness may be associated with signal transduction at the proteomic level rather than at the transcriptomic level. Cells treated with metformin also showed a statistically significant reduction of *IRS‐1* expression after 2 h of treatment, similar to spexin‐treated cells. These results differ from several studies which reported increased *IRS‐1* expression following metformin treatment in rat muscles (4 weeks) and L6 myotubes (24 h) [65, 66]. Interestingly, metformin treatment did not alter the *IRS‐1* expression in rat liver (4 week treatment) but significantly decreased its levels in human luteinized granulosa cells (24 h treatment) [67, 68]. These findings imply that the effects of metformin on *IRS‐1* expression may vary depending on the experimental model used and treatment duration.

For spexin‐treated C2C12 myotubes, *PI3K* expression decreased initially at 1 and 2 h compared with the control group but remained upregulated after 4 and 24 h (Figure 5). The effects of spexin on *PI3K* expression have not been reported previously in the literature to date. The present findings seem to be consistent with other research which revealed that N1‐methylnicotinamide (MNAM) treatment did not significantly affect *PI3K* protein level in insulin‐resistant C2C12 myocytes but alleviated insulin resistance by promoting phosphorylation of *PI3K* [69]. Likewise, metformin treatment in this study did not significantly affect *PI3K* expression. However, a previous study reported that metformin treatment under the same conditions upregulated *PI3K* expression [70]. Notably, that study included a cotreatment with 100 nM insulin, suggesting the effects of metformin may be enhanced by insulin [70]. Hence, further investigation is required to determine whether insulin co‐administration enhances metformin's effects on *PI3K* expression and its underlying mechanisms.

Treatment of C2C12 myotubes with 1000 nM spexin resulted in an initial downregulation of *GLUT4* expression at 1 h, followed by upregulation at 2, 4, and 24 h (Figure 6). This accords with earlier observation, which showed that 400 nM and 800 nM spexin significantly upregulated *GLUT4* expression in L6 myotubes after 12 h of treatment [33]. This outcome may be attributed to differences in experimental conditions, such as cell lines, treatment durations, or type of treatment and concentrations, which could account for the discrepancies observed. Results from metformin‐treated C2C12 myotubes did not significantly increase the *GLUT4* expression, even after 24 h of treatment. The present findings seem to be consistent with other research which found 48 h of 1 mM metformin treatment did not alter the protein expression of *GLUT4* in the total cell lysates of C2C12 myotubes but increased its membrane localization and glucose uptake [71]. However, a 48 h treatment with 1.5 mM metformin increased *GLUT4* gene expression in palmitate and tumor necrosis factor‐α (*TNFα*)‐induced insulin‐resistant C2C12 myotubes [72]. These results suggest that the metformin treatment used in this study is suboptimal, in which a higher metformin concentration and longer treatment duration may be necessary to upregulate *GLUT4* expression.

Spexin, a peptide hormone, holds significant potential in diabetes research, as peptides have been extensively studied for their roles in managing T2DM. For instance, pentapeptides LPLLR, derived from walnut protein hydrolysates, and KIWHHTF from sturgeon protein hydrolysates improved glucose uptake in insulin‐resistant HepG2 hepatocytes by upregulating the phosphorylation of *IRS‐1*, *PI3K*, and *Akt* [73]. Ginseng peptides yielded similar effects in the liver of a diabetic mouse model, which significantly improved glycaemic control [74]. WL15 peptide obtained from *Channa striatus* increased the expression levels of insulin receptor tyrosine kinase (*IRTK*), *IRS‐1*, *PI3K*, and *GLUT4*, resulting in enhanced glucose uptake in high glucose‐treated L6 myotubes [75]. Overall, these findings suggest that these peptides ameliorate insulin resistance by activating the *IRS‐1*/*PI3K*/Akt pathway.

Contrary to expectations, spexin treatment on insulin‐resistant C2C12 myotubes in this study did not exert similar changes in the gene expression levels although spexin had shown hypoglycaemic effects in a previous study [33]. Notably, although changes in gene expression of *IRS‐1*, *PI3K*, and *GLUT4* were non‐significant at most time points, the overall pattern of the effect of spexin on these gene levels has closely resembled metformin. These findings suggest that both compounds may exert their hypoglycaemic effects through a similar mechanism of action. A comparable outcome was observed with *Cassia abbreviata* seed extract, which downregulated the gene expression of *IRS‐1*, *PI3K*, and *GLUT4* yet significantly promoted glucose uptake in C2C12 cells [76]. These findings corroborate the use of spexin may mediate glucose uptake by stimulating *GLUT4* translocation without requiring the synthesis of additional proteins of the insulin‐dependent pathway. The underlying mechanism may involve activating existing proteins or reliance on an alternative pathway such as the insulin‐independent pathway to facilitate *GLUT4* translocation and increase glucose uptake.

Overall, our findings suggest that spexin exposure under high‐glucose conditions is associated with specific changes in IRS‐1, PI3K, GLUT4, and PGC‐1α mRNA expression in C2C12 myotubes. A limitation of this study is that insulin resistance in our high‐glucose C2C12 model was not directly confirmed by acute insulin stimulation (10–30 min) with assessment of phosphorylated‐AKT, phosphorylated‐IRS, or glucose uptake. Hence, these results would be interpreted as changes in insulin‐related gene expression under high‐glucose and spexin treatment, rather than definitive evidence of altered insulin resistance or insulin sensitivity. Furthermore, our analyses were restricted to mRNA levels. Future studies incorporating protein‐level and functional assays as well as in vivo models will be necessary to determine whether these transcriptional changes translate into meaningful alterations in insulin signaling and to further elucidate the mechanistic actions of spexin under hyperglycaemic conditions.

## 5 Conclusion

This study shows that high glucose and spexin treatment alter the expression of insulin‐signaling–related genes in C2C12 myotubes, supporting a potential link between spexin and the regulation of insulin pathway components at the transcriptional level. Spexin, known for its role in glucose homeostasis, was assessed for its effects on *IRS‐1*, *PI3* and *GLUT4* gene expression in C2C12 myotubes cultured in high‐glucose condition. C2C12 myoblasts differentiation was confirmed, and spexin was found to be non‐cytotoxic at concentrations up to 1000 nM. High‐glucose incubation did not affect myogenesis but was associated with statistically significant downregulation of *PGC‐1α*, while GLUT4 and HKII were modestly decreased without statistical significance, consistent with transcriptional changes often observed in insulin‐resistant skeletal muscle. Spexin downregulated *IRS‐1* at 2 h but had no significant effect on *PI3K* or *GLUT4* expression, with findings comparable to metformin. These results suggest that spexin does not regulate glucose homeostasis via these genes and may act through alternative mechanisms, warranting further investigation. Taken together, these results provide early, transcriptional‐level evidence and form a basis for future in vivo and mechanistic studies, including protein‐level validation and reverse manipulation of spexin receptors, to elucidate how spexin affects skeletal muscle insulin signaling in the setting of hyperglycaemia.

## Author Contributions

A.S.C.: data curation, formal analysis, investigation, methodology, writing original draft, review and editing. J.W.T.: data curation, formal analysis, methodology, resources, supervision, validation, writing review and editing. F.J.: data curation, formal analysis, methodology, resources, supervision, validation, writing review and editing. W.W.Y.Y.: conceptualization, data curation, formal analysis, funding acquisition, methodology, project administration, resources, supervision, validation, writing review and editing.

## Funding

This work was supported by the Early Career Research (ECR) Grant Scheme 2023, Monash University Malaysia [I‐M010‐ECR‐000055] and the Incubator Grant 2024, Monash University [P‐M010‐CNI‐000187].

## Ethics Statement

Ethical approval was not required for this study as it involved the use of commercially available cell lines only and did not include human participants or animal subjects. The C2C12 cell line was purchased from American Type Culture Collection (ATCC), U.S.

## Conflicts of Interest

The authors declare no conflicts of interest.

## Supporting information


**Appendix 1** (A) Experimental protocol. Myoblast differentiation into myotubes was initiated by culturing C2C12 cells in a growth medium supplemented with 10% fetal bovine serum (FBS) for 3 days (Day −3 to Day −1). On the following day (Day 0), the medium was replaced with a differentiation medium, where 10% FBS was substituted with 2% horse serum to promote cell differentiation (B) Analysis of differentiation progression in C2C12 cells with H&E staining and fusion index quantification. Representative bright‐field images of H&E‐stained cells were taken under the same magnification (x200) at various time points. (i) Day 0 (pre‐differentiation); (ii) Day 1; (iii) Day 3; (iv) Day 6. Scale bar, 200 μm. The arrows indicate the multinucleated myotubes whereas the arrowheads indicate the unfused myoblasts. (v) Fusion index quantification (in %) based on H&E staining of C2C12 cells at the corresponding timepoints. Values were represented as mean ± SEM (*n* = 3). Significance compared to day 0 was evaluated with a one‐way ANOVA test and a follow‐up Tukey test. ***, *p* < 0.005 (C) PCR analysis of differentiation markers in undifferentiated myoblasts (Day 0) and differentiated myotubes (Day 6).

## Data Availability

Stored in repository.
