# Supplementary figures and images for: Investigation of Spexin Effects on Insulin‐Dependent Pathway Genes in High‐Glucose‐Induced Insulin‐Resistant C2C12 Myotubes
Source: FASEB Bioadv. 2026 Jun 23;8(6):e70131. doi: 10.1096/fba.2026-00003 (PMC13288056; doi:10.1096/fba.2026-00003)

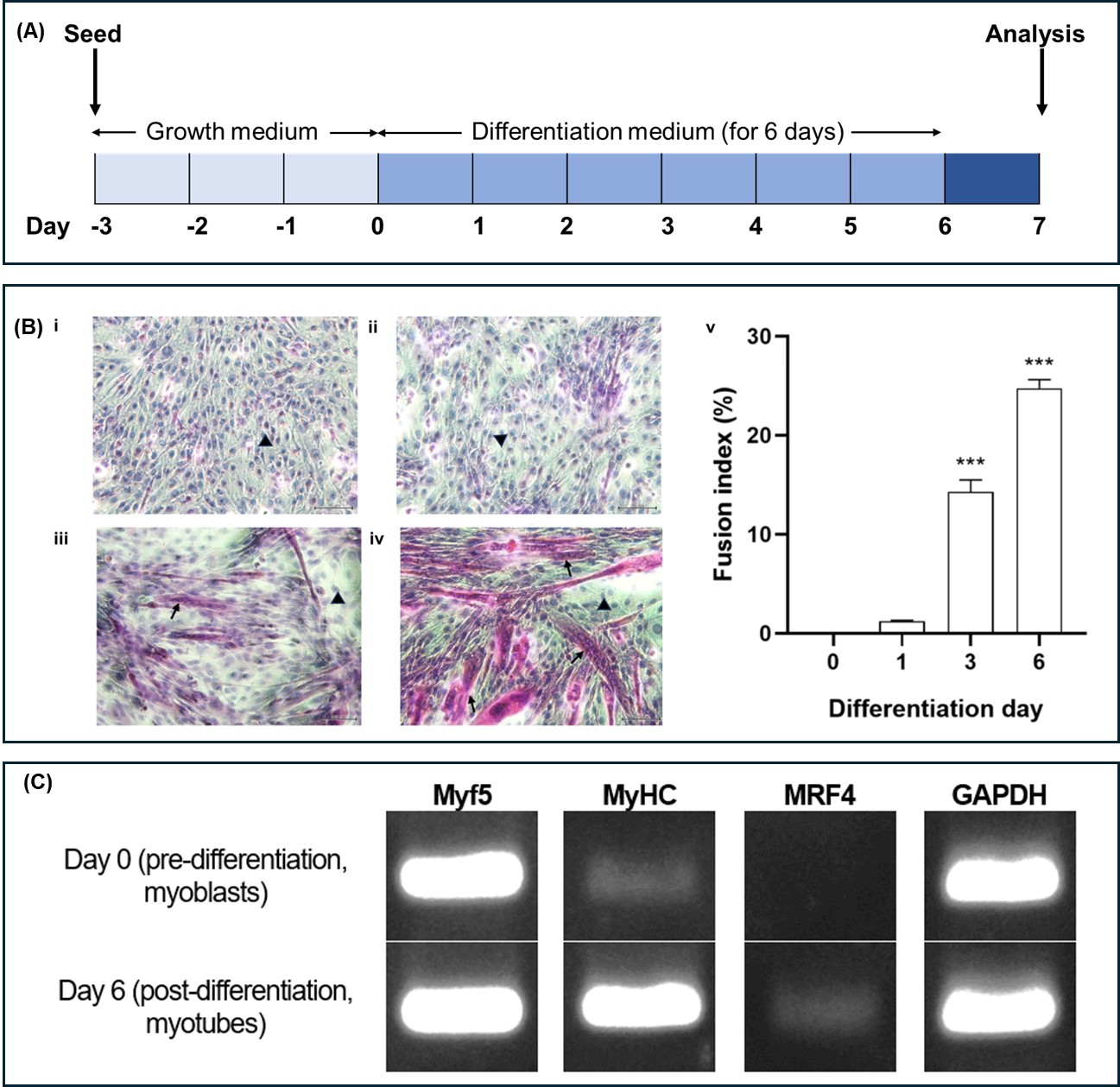

Supplement: Supplementary file 1 — Appendix 1 (A) Experimental protocol. Myoblast differentiation into myotubes was initiated by culturing C2C12 cells in a growth medium supplemented with 10% fetal bovine serum (FBS) for 3 days (Day −3 to Day −1). On the following day (Day 0), the medium was replaced with a differentiation medium, where 10% FBS was substituted with 2% horse serum to promote cell differentiation (B) Analysis of differentiation progression in C2C12 cells with H&E staining and fusion index quantification. Representative bright‐field images of H&E‐stained cells were taken under the same magnification (x200) at various time points. (i) Day 0 (pre‐differentiation); (ii) Day 1; (iii) Day 3; (iv) Day 6. Scale bar, 200 μm. The arrows indicate the multinucleated myotubes whereas the arrowheads indicate the unfused myoblasts. (v) Fusion index quantification (in %) based on H&E staining of C2C12 cells at the corresponding timepoints. Values were represented as mean ± SEM (n = 3). Significance compared to day 0 was evaluated with a one‐way ANOVA test and a follow‐up Tukey test. ***, p < 0.005 (C) PCR analysis of differentiation markers in undifferentiated myoblasts (Day 0) and differentiated myotubes (Day 6). [file FBA2-8-e70131-s001.jpg]
